# Impact of virulence factors overexpression on *Listeria monocytogenes* F2365 epidemic strain fitness and the limitations of surrogate species in UHT and raw milk

**DOI:** 10.1038/s41538-025-00658-7

**Published:** 2025-12-15

**Authors:** Alba Espí-Malillos, Inmaculada López-Almela, Pilar Ruiz-García, María Carmen López-Mendoza, Nerea Carrón, Pedro González-Torres, Jazmin Meza-Torres, Javier Pizarro-Cerdá, Juan J. Quereda

**Affiliations:** 1 Grupo de investigación LisBio, Departamento Producción y Sanidad Animal, Salud Pública Veterinaria y Ciencia y Tecnología de los Alimentos, Facultad de Veterinaria, Universidad Cardenal Herrera-CEU, CEU Universities, 46115 Valencia, España; 2 Departamento de Ciencias Biomédicas, Facultad de Ciencias de la Salud, Universidad Cardenal Herrera-CEU, CEU Universities, 46115 Valencia, España; 3 Departamento Producción y Sanidad Animal, Salud Pública Veterinaria y Ciencia y Tecnología de los Alimentos, Facultad de Veterinaria, Universidad Cardenal Herrera-CEU, CEU Universities, 46115 Valencia, España; 4 Microomics Systems S.L., 08041 Barcelona, España; 5 Sir William Dunn School of Pathology, University of Oxford, Oxford, OX1 3RE UK; 6 Institut Pasteur, Université Paris Cité, Yersinia Research Unit, WHO Collaborating Research and Reference Centre for Plague FRA-146, Paris, France

**Keywords:** Diseases, Microbiology

## Abstract

Identifying reliable surrogates for *Listeria monocytogenes* is critical to safely model its behavior in dairy environments. Many dairy-related listeriosis outbreaks are linked to unpasteurized milk products enriched in hypervirulent *L. monocytogenes* from lineage I. We used an outbreak-associated lineage I strain to evaluate *Listeria innocua*, *Listeria valentina*, and *Listeria ivanovii* as potential surrogates in UHT and raw milk at 4 °C. We also assessed how overexpression of key virulence factors, including LIPI-3 and the PrfA regulon, influenced growth. While LIPI-3 overexpression had no significant impact, PrfA overexpression reduced fitness in both matrices. None of the other *Listeria* species tested accurately replicated the *L. monocytogenes* wild type strain growth patterns. Additionally, the native raw milk microbiota remained largely unaffected by the presence of any *Listeria* strain. Our findings emphasize the limitations of commonly used surrogates and underline the importance of selecting appropriate models for food challenge studies, especially in complex dairy matrices.

## Introduction

The genus *Listeria* is composed of 29 species^1^. Only two *Listeria* spp. are considered pathogenic within the genus: *L. monocytogenes* and *L. ivanovii*^2^. *L. ivanovii* affects ruminants and rarely causes infections in humans^3^, whilst *L. monocytogenes* causes illness in humans and other animals, mainly domestic ruminants^4^. Listeriosis accounted for the highest case fatality rate (19.7%) among all reported foodborne diseases^5^.

The most studied species is *L. monocytogenes*, a ubiquitous, saprophytic, and psychrophilic bacterium that can survive and grow in different environments, including water, soil, sewage, vegetation, animal feeds, farms, food products, and food-processing settings^4^. It is also a facultative intracellular pathogen that causes foodborne listeriosis^6^. After the ingestion of contaminated food, *L. monocytogenes* can cross the intestinal barrier, disseminate via the bloodstream, and reach the central nervous system and fetus, causing septicemia, meningoencephalitis, and abortion^6,7^.

*L. monocytogenes* can be divided into four evolutionary lineages and more than 170 clonal complexes (CCs)^8^. While lineage I isolates, in particular CC1, CC4, and CC6, are hypervirulent and associated with human and animal clinical cases, lineage II, in particular CC9 and CC121, is mainly associated with food and food processing environments^9^. Importantly, epidemiological studies have shown that hypovirulent isolates of CC9 and CC121 are strongly associated with meat products, whereas hypervirulent CC1, CC4, and CC6 are associated with dairy products^10^. Moreover, dairy products are involved with nearly half of the registered listeriosis outbreaks in the United States and Europe^11–13^.

*L. monocytogenes* virulence depends mainly on *Listeria* pathogenicity islands (LIPIs) LIPI-1, LIPI-3, LIPI-4, and the internalin A and B loci^8^. PrfA is the major virulence regulator in *L. monocytogenes* and controls the internalin A and B loci for attachment to and invasion of the intestinal epithelium and LIPI-1, which is required for intracellular growth and transmission from cell to cell^4,8^. Although not common, there are atypical *L. monocytogenes* strains that display a PrfA* mutation leading to constitutive production of LIPI-1 virulence genes (e.g. the lineage II EGD strain)^14,15^. The internalin A and B locus and LIPI-1 genes are present in all *L. monocytogenes* strains^8^. However, LIPI-3 (which encodes the bacteriocin listeriolysin S (LLS) required for intestinal colonization) and LIPI-4 (involved in brain and placental invasion) are only present in some hypervirulent lineage I clonal complexes^9,16^.

The saprophytic/extracellular and the infective/intracellular facets of *L. monocytogenes* are complementary and have frequently been studied independently. However, there are cases such as ActA, a virulence factor encoded in LIPI-1 and regulated by PrfA, that plays a critical role during infection and the extracellular lifestyle of *L. monocytogenes*^17^. During the intracellular cycle, ActA contributes to *L. monocytogenes* actin-based motility, cell-to-cell spread, and dissemination within host tissues^17^. ActA also acts extracellularly, mediating *L. monocytogenes* aggregation and biofilm formation^17^. The consequences of the contamination of a dairy product with a hypervirulent strain of *L. monocytogenes* with high PrfA or LIPI-3 activity are currently unknown. This lack of knowledge led us to investigate the role that PrfA-controlled genes and LIPI-3 could play in food contamination and growth.

Understanding how *L. monocytogenes* grows and survives in food is essential for proposing practical strategies that prevent its transmission. Food industries try to identify possible scenarios that represent good indicators of conditions in which *L. monocytogenes* can thrive. The identification of bacteria belonging to the genus *Listeria* has been used as an indicator to detect conditions that allow for the presence, growth, and persistence of *L. monocytogenes*^18^. Due to *L. monocytogenes* pathogenicity, modeling and understanding its behavior in foods and food processing environments could require using a surrogate organism. *L. innocua* is a closely related, generally non-pathogenic species of the *sensu stricto* group traditionally used as a surrogate organism to better understand the behavior of *L. monocytogenes* in different environments^19^. The use of *L. innocua* to predict the behavior of *L. monocytogenes* in agricultural and food processing settings has historically been justified by the two species’ ecological cohabitation, genomic closeness, and physiological resemblance^20^. *L. monocytogenes*, *L. innocua*, and *L. ivanovii* belong to the *Listeria sensu stricto* clade, and therefore share phylogenetic proximity and ecological traits^21,22^. These three species are the most well characterized among the *Listeria* genus^4,22^. Despite the genetic closeness of these three species, the use of *L. ivanovii* and *L. innocua* as surrogates in dairy products lacks extensive experimental evidence*. L. valentina* belongs to the *Listeria sensu lato* clade and was recently described in a dairy farm^23^. However, the use of *L. valentina* as a surrogate has never been tested before.

Here, using the lineage I strain F2365 (responsible for the 1985 California outbreak related to Mexican fresh cheese, one of the deadliest bacterial foodborne outbreaks ever reported in the United States^11^ we first analyzed the reliability of *L. innocua*, *L. valentina*, or *L. ivanovii* as surrogates for the pathogenic *L. monocytogenes* to monitor growth in Ultra-High-Temperature (UHT) and raw milk at refrigeration temperatures. Secondly, we determined the extent to which virulence factors such as the bacteriocin LLS encoded in LIPI-3 or those virulence factors controlled by PrfA contribute to *L. monocytogenes* growth in UHT and raw milk at refrigeration temperatures. Finally, we analyzed the impact of milk contamination with *L. innocua*, *L. valentina*, *L. ivanovii*, *L. monocytogenes* F2365 or its isogenic mutants overexpressing PrfA and LIPI-3 on the growth and composition of the native raw milk microbiota by using growth kinetics studies and metagenomic analysis.

## Results

The growth of *L. monocytogenes* strains F2365, F2365_PrfA*, and F2365_LLS and *L. innocua*, *L. ivanovii*, and *L. valentina* strains was investigated at 4 °C at an inoculum level of 200 CFU/mL in UHT milk for 43 days and raw milk for 35 days. Additionally, the pH of the milk in the inoculated tubes was measured throughout the storage period.

### Growth of non-pathogenic *Listeria* spp., *L. ivanovii*, and epidemic *L. monocytogenes* overexpressing virulence determinants in UHT milk at refrigerated temperature

**Fig. 1:**
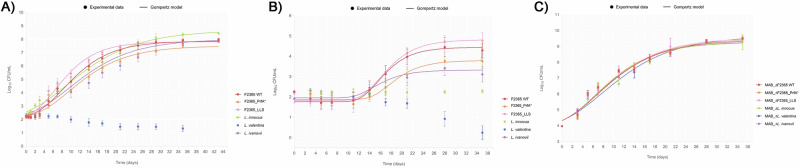
Growth dynamics of *Listeria* species and mesophilic aerobic bacteria in milk at 4 °C. Growth of *Listeria* spp. was monitored in UHT milk (A) and raw milk (B), and mesophilic aerobic bacteria (MAB) was assessed in raw milk (C) at 4 °C. *Listeria* spp. counts were enumerated at 0, 0.5, 1, 2, 3, 5, 7, 10, 14, 17, 21, 25, 29, 35, and 43 days in UHT milk. *Listeria* spp., and MAB counts were enumerated at 0, 3, 5, 7, 11, 14, 17, 21, 28, and 35 days in raw milk. The error bars showing standard deviations were performed based on four biological replicates for each strain and type of milk.

All *L. monocytogenes* isolates, *L. innocua*, and *L. ivanovii* grew in UHT milk at 4 °C after a storage period of 1-2 days and reached a maximal population density between 7.50 and 8.00 Log_10_ CFU/mL after 28 days (Fig. 1A). In contrast, *L. valentina* did not grow under the tested conditions and showed a progressive decline in CFU/mL after day 7 (Fig. 1A). When *L. monocytogenes* isolates, *L. ivanovii*, and *L. innocua* were individually inoculated into UHT milk, statistically significant differences in lag phase (λ), maximal growth rate (µ_max_), and maximal population density were observed among the strains during the 43 days of the study (*P* < 0.05; Table 1). *L. innocua* exhibited a significantly shorter λ in UHT milk compared to both *L. monocytogenes* F2365 and F2365_PrfA* (*P* < 0.05) (Fig. 1A, Table 1). Additionally, the F2365_PrfA*, *L. innocua* and *L. ivanovii* showed significantly lower μ_max_ than F2365_LLS and *L. monocytogenes* F2365 (*P* < 0.05) (Table 1). *L. innocua* reached the highest maximal population density in UHT milk at 4 °C, which was comparable among all strains, except for *L. monocytogenes* F2365_PrfA*, which reached a significantly lower maximal population density (Fig. 1A, Table 1).

**Table 1:** Estimated kinetic parameters of *Listeria* spp. grown in milk at 4 °C obtained from the modified Gompertz model

Kinetic parameter	Milk type	*Listeria* spp. strain					
		*Lm* F2365	*Lm* F2365_PrfA*	*Lm* F2365_LLS	*L. innocua*	*L. valentina*	*L. ivanovii*
λ (days)	UHT milk	2.29 ± 0.62a	2.42 ± 0.33a	1.42 ± 0.18 ab	0.00 ± 0.00b	- -	1.47 ± 0.60 ab
	raw milk	12.21 ± 1.45ab	14.17 ± 1.13a	12.25 ± 0.76 ab	--	--	11.17 ± 0.12 b
µ_max_ [Log_10_(CFU/mL)/day]	UHT milk	0.35 ± 0.03 a	0.28 ± 0.01b	0.41 ± 0.02a	0.27 ± 0.04b	--	0.26 ± 0.04 b
	raw milk	0.30 ± 0.03 a	0.21 ± 0.03b	0.30 ± 0.03a	- -	- -	0.15 ± 0.04b
Log_10_Nf_max_ [Log_10_(CFU/mL)]	UHT milk	7.86 ± 0.04ab	7.50 ± 0.11 a	7.81 ± 0.09ab	8.66 ± 0.04b	- -	8.00 ± 0.22 ab
	raw milk	4.44 ± 0.50ab	3.79 ± 0.38ab	4.81 ± 0.43a	--	--	3.32 ± 0.69b
RMSE	UHT milk	0.259	0.263	0.315	0.275	-	0.263
	raw milk	0.157	0.184	0.135	-	-	0.198
R^2^adj.	UHT milk	0.985	0.982	0.979	0.981	-	0.983
	raw milk	0.980	0.953	0.989	-	-	0.860

Data represents estimated parameter ± standard deviation. Values in the same row followed by the same lowercase letter are not significantly different (*P* > 0.05). Parameters are estimated from the growth curves and each point of the curves was based on four replicate samples in UHT milk and raw milk.

At the beginning of the experiment, UHT milk had a typical pH of 6.82, and no significant changes in pH were observed throughout the monitored period (Table S1). No correlation was found between bacterial growth (Log_10_CFU/mL) and pH values for any of the strains tested (Table S1).

Altogether, these results indicate that the *L. innocua, L. valentina* and *L. ivanovii* isolates tested are non-reliable surrogates for monitoring *Listeria monocytogenes* growth in UHT milk stored at 4 °C. Furthermore, the reduced growth rate of the PrfA* mutant shows a fitness cost compared to the parental strain in UHT milk at 4 °C (Fig. 1A, Table 1).

### Growth of non-pathogenic *Listeria* spp., *L. ivanovii*, and epidemic *L. monocytogenes* overexpressing virulence determinants in raw milk at refrigerated temperature

All *L. monocytogenes* isolates and *L. ivanovii* grew in raw milk at 4 °C after a storage period ranging from 11 to 14 days and reached a maximal population density between 3.32 and 4.81 Log₁₀ CFU/mL after 28 days (Fig. 1B, Table 1). *L. innocua* did not grow, but its population levels remained stable throughout the 35-day study (Fig. 1B). In contrast, *L. valentina* failed to grow and showed a progressive decline in CFU/mL counts after day 14 (Fig. 1B). When *L. monocytogenes* isolates and *L. ivanovii* were individually inoculated into raw milk, statistically significant differences in lag phase (λ), maximal growth rate (µ_max_), and maximal population density were observed among the strains during the 35 days of the study (*P* < 0.05) (Table 1). Among all strains tested *L. monocytogenes* F2365_PrfA* displayed the longest adaptation period, with a significantly longer λ than *L. ivanovii*, which adapted most rapidly to raw milk at 4 °C (*P* < 0.05) (Fig. 1B, Table 1). Both *L. monocytogenes* F2365_PrfA* and *L. ivanovii* showed impaired growth rates in raw milk, with significantly lower μ_max_ values compared to *L. monocytogenes* F2365 and F2365_LLS strains (*P* < 0.05) (Table 1). Regarding *L. monocytogenes* and *L. ivanovii* isolates, *L. monocytogenes* F2365_LLS and *L. ivanovii* reached the maximal and minimal population density, respectively (*P* < 0.05) (Table 1 and Fig. 1B). Although not statistically significant, *L. monocytogenes* F2365_PrfA* showed a lower maximal population density than *L. monocytogenes* F2365.

At the beginning of the experiment, raw milk had a typical pH of 6.86, and no significant changes in pH were observed throughout the monitored period (Table S2). No correlation was found between bacterial growth (Log_10_CFU/mL) and pH values for any of the strains tested (Table S2).

Altogether, these results indicate that *L. ivanovii*, *L. innocua*, and *L. valentina* are not suitable surrogates for the F2365 *L. monocytogenes* epidemic strain in monitoring growth in raw milk at refrigeration temperatures. PrfA* overexpression resulted in a significant fitness cost; in contrast, LLS constitutive expression did not significantly affect growth parameters compared to the wild type strain during competition with the native microbiota of raw milk (Fig. 1B, Table 1).

### Growth of mesophilic aerobic bacteria in raw milk at refrigerated temperature

The initial mesophilic aerobic bacteria (MAB) population density on raw milk samples at the beginning of the study were 3.97 Log_10_CFU/mL (Fig. 1C, Table S3). MAB increased between 9.12 and 9.65 Log_10_CFU/mL after 35 days of storage at 4 °C in all inoculated samples (Fig. 1C, Table S3). No statistically significant differences were detected for λ, μ_max_, and the maximal final concentration of MAB in raw milk contaminated with the different *Listeria* spp. isolates tested (*P* > 0.05) (Table S3).

Altogether, these results showed that the pathogenic properties of the *Listeria* spp. inoculated do not influence the kinetic parameters of MAB populations of raw milk.

### Effect of contamination of non-pathogenic *Listeria* spp., *L. ivanovii*, and epidemic *L. monocytogenes* overexpressing virulence determinants on the bacterial microbiome of raw milk at 4 °C

**Fig. 2:**
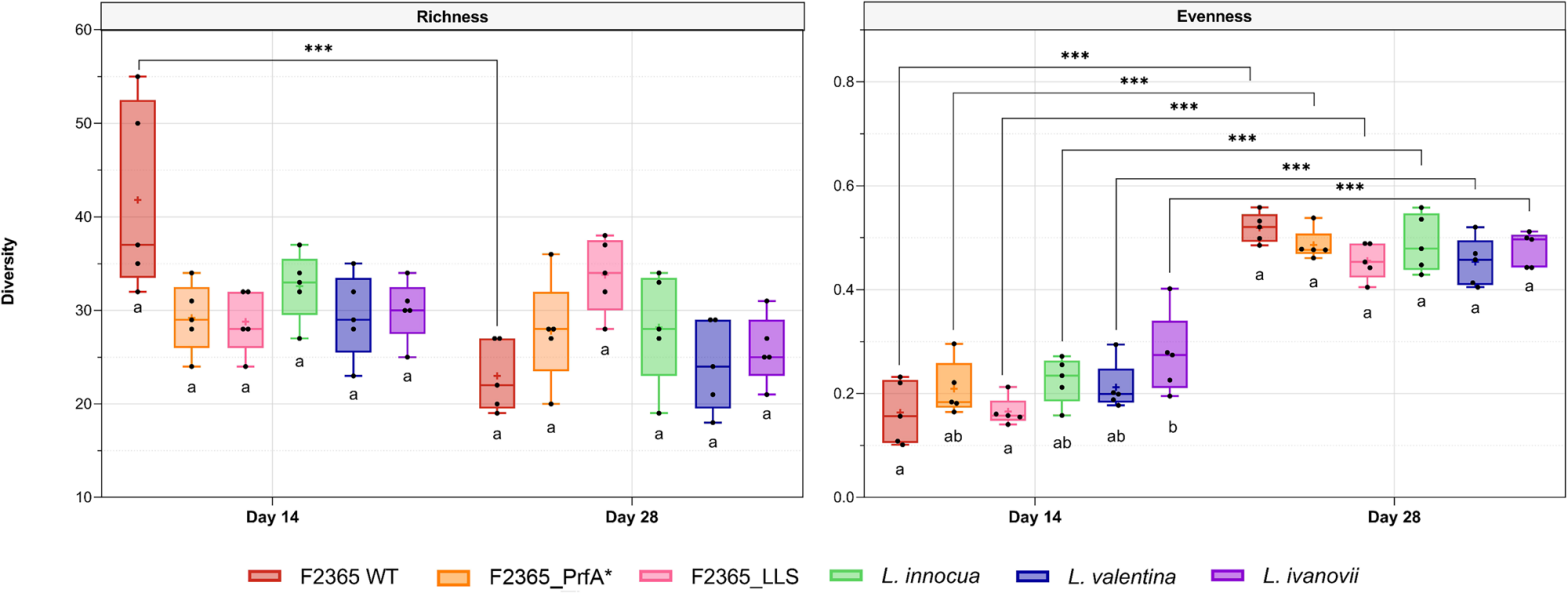
Alpha diversity of raw milk samples. Boxplots showing the differences in alpha diversity measured as Richness, meaning the number of observed ASVs (amplicon sequence variants), and Evenness, regarding the abundance of these ASVs, according to *L. monocytogenes* F2365 wild type (WT), its constitutively active PrfA mutant (PrfA*), and the listeriolysin S mutant (LLS), together with the non-pathogenic species *L. innocua*, *L. valentina*, and *L. ivanovii*. Differences between groups were calculated by ANOVA based on estimated marginal means. *P < 0.05; **P > 0.01; ***P < 0.001.

The Richness of microbial communities on raw milk samples inoculated with *L. monocytogenes* F2365 was significantly reduced at day 28 when compared to day 14 (*P* ≤ 0.001) (Fig. 2). The Richness of microbial communities on raw milk samples inoculated with the rest of *Listeria* spp. was not significantly reduced at day 28 when compared to day 14 (*P* > 0.05) (Fig. 2).

Independently of the *Listeria* spp. inoculated into the raw milk, Evenness at day 28 was significantly higher than at day 14 (*P* ≤ 0.001) (Fig. 2). Although raw milk samples inoculated with *L. ivanovii* showed significantly higher Evenness than those inoculated with *L. monocytogenes* F2365 WT and F2365_LLS at day 14 (*P* ≤ 0.001), those differences were not observed at day 28 (*P* > 0.05) (Fig. 2).

**Fig. 3:**
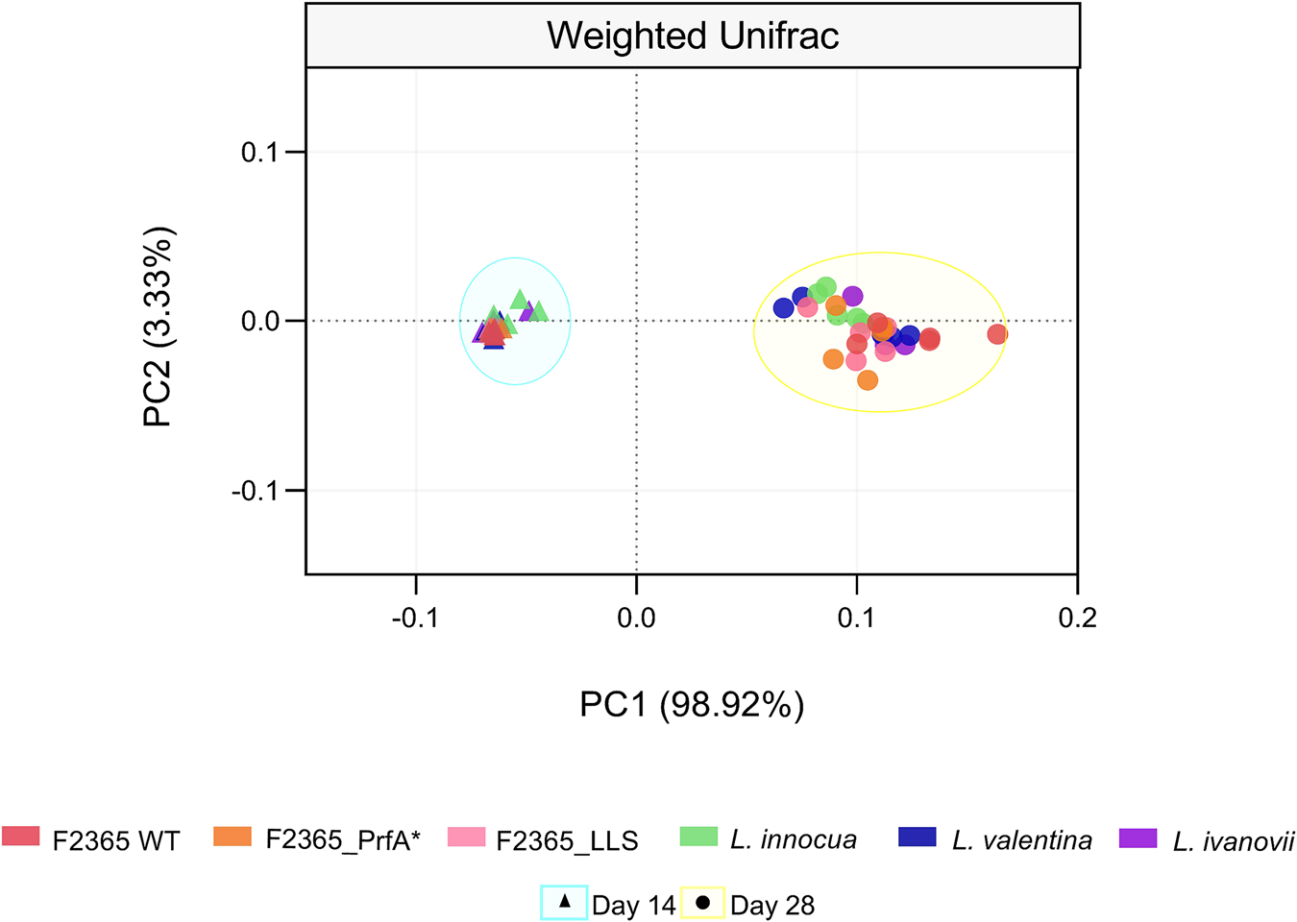
Beta diversity of raw milk samples. Principal coordinates analysis (PCoA) based on Weighted UniFrac distances shows the structure of raw milk bacterial communities inoculated with *Listeria monocytogenes* F2365 wild type (WT), its constitutively active PrfA mutant (PrfA*), and the listeriolysin S mutant (LLS), together with the non-pathogenic species *L. innocua*, *L. valentina*, and *L. ivanovii*. Bacterial communities are denoted with distinct symbols for day 14 (triangles), and for day 28 (circles). Each point corresponds to one of the five biological replicates for each raw milk sample.

The PCoA based on Weighted UniFrac analysis revealed that the majority of the differences in microbial composition were attributed to time-related alterations in the native microbiota, as indicated by the separation along PC1, which accounted for 98.92% of the variance (*P* ≤ 0.05) (Fig. 3). The PCoA findings indicated that samples collected on day 14 demonstrated higher similarity with limited variation. In contrast, samples from day 28 displayed more significant variability (Fig. 3). Comparisons within day 14 revealed differences between raw milk samples inoculated with *L. monocytogenes* F2365 and *L. innocua*, *L. monocytogenes* F2365 and *L. ivanovii*, *F2365*_LLS and *L. ivanovii*, and F2365_PrfA* and *L. ivanovii* (*P* ≤ 0.05) (Fig. 3). At day 28, significant differences were also detected between raw milk samples inoculated with *L. monocytogenes* F2365 and *L. innocua* (*P* ≤ 0.05) (Fig. 3). Altogether, these findings indicate that temporal progression was the dominant factor shaping the microbial communities in raw milk.

**Fig. 4:**
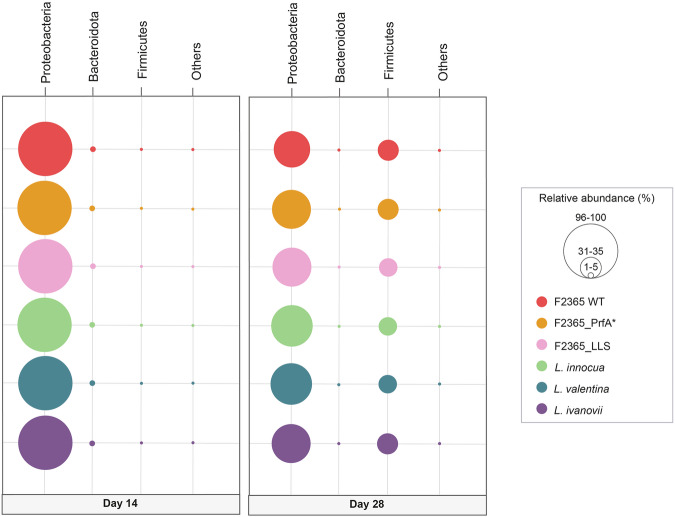
Phyla of raw milk samples. Bubble plots showing the composition of the raw milk microbiota population inoculated with *L. monocytogenes* F2365 wild type (WT), its constitutively active PrfA mutant (PrfA*), and the listeriolysin S mutant (LLS), together with the non-pathogenic species *L. innocua*, *L. valentina*, and *L. ivanovii*. Phyla with a relative abundance of less than 1% have been grouped as “Other”.

On both days 14 and 28 of the study, the microbial communities in raw milk were primarily composed of the Proteobacteria phylum (>97% in all samples at day 14), regardless of the specific *Listeria* species present (Fig. 4). On day 14, the second most prevalent phylum was Bacteroidota (Fig. 4). Regardless of the inoculated *Listeria* species, Firmicutes exhibited a significant increase in relative abundance by day 28, becoming the second most prevalent phylum in the microbial community (Fig. 4).

**Fig. 5:**
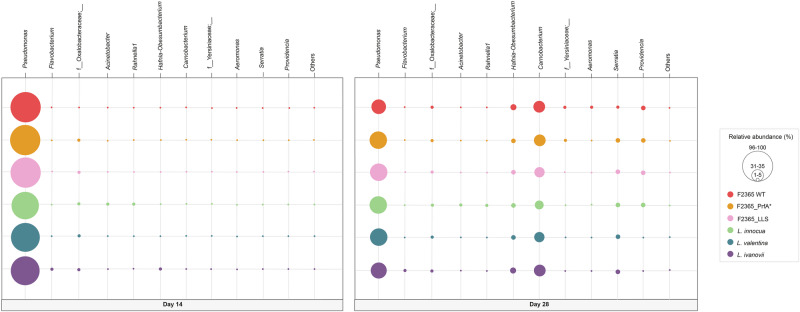
Genus of raw milk samples. Bubble plots showing the composition of the raw milk microbiota population inoculated with *L. monocytogenes* F2365 wild type (WT), its constitutively active PrfA mutant (PrfA*), and the listeriolysin S mutant (LLS), together with the non-pathogenic species *L. innocua*, *L. valentina*, and *L. ivanovii*. Genera with a relative abundance of less than 1% have been grouped as “Other”.

On days 14 and 28 of the study, the microbial communities in raw milk were primarily dominated by the *Pseudomonas* genus (more than 90% relative abundance in all groups on day 14, regardless of the inoculated *Listeria* spp. (Fig. 5)). On day 14, genera belonging to f_*Oxalobacteraceae* were the second most prevalent (Fig. 5). In contrast, on day 28, *Carnobacterium* increased its relative abundance and became the second most prevalent genus, irrespective of the *Listeria* spp. present in the raw milk (Fig. 5). Moreover, there were significant increases in the relative abundances of *Hafnia-Obesumbacterium* and *Serratia* in samples inoculated with all the *Listeria* strains used in this study (Fig. 5). A significant increase in the relative abundance of *Providencia* was also observed at day 28 in samples inoculated with *L. monocytogenes*, *L. monocytogenes*_LLS, *L. monocytogenes*_PrfA*, and *L. innocua* (Fig. 5).

Overall, these data indicate that the overexpression of virulence traits of the contaminating *L. monocytogenes* F2365 or the specific characteristics of the non-pathogenic *Listeria* species did not influence the dynamics of microbial communities in raw milk.

## Discussion

Comprehensive approaches must be implemented when modeling and predicting the growth of *L. monocytogenes* in dairy products made of milk under refrigeration conditions. To our knowledge, apart from the present study, only two studies have previously evaluated the growth potential of the low number of *Listeria* spp. cells frequently counted in raw milk^24,25^. In the present study, lower CFU counts were observed for *Listeria* spp. in raw milk compared to UHT milk (Fig. 1A, B), showing that native microbiota can limit the growth of *Listeria* spp. This inhibitory effect aligns with previous studies of our group where raw milk also restricted *L. monocytogenes* growth under cold storage^25^. Notably, this growth limitation contrasts with the homogenous growth of MAB CFU across the study (Fig. 1C).

In food safety studies, *L. innocua* is commonly used as a non-pathogenic surrogate for *L. monocytogenes*^26–32^. However, our findings indicate that *L. innocua* growth in raw milk microbiota at refrigeration temperatures is restricted. This result suggests that *L. innocua* may not always be a reliable model for *L. monocytogenes* survival or growth, particularly in complex microbial environments such as refrigerated dairy products made of raw milk. Previous studies have shown that certain *L. monocytogenes* strains are more resistant to cold stress than *L. innocua*^33,34^, and variations in *L. innocua’s* thermal resistance^26^. further complicate its use as a universal indicator. Our findings indicate that *L. innocua* or *L. ivanovii* would not be adequate surrogates in milk at refrigeration temperatures. Finally, *L. valentina* is not a reliable surrogate for *L. monocytogenes* in UHT or raw milk at refrigeration temperatures, as it did not grow under these conditions. The present results highlight the need for a more precise selection of surrogate organisms tailored to specific food matrices and environmental conditions, ensuring an accurate risk assessment. Moreover, different surrogates may be required depending on the *L. monocytogenes* lineage to be evaluated, such as host-adapted lineage I (hypervirulent) or food-adapted lineage II (hypovirulent)^9,20^.

Most dairy-related outbreaks have been linked to products made of unpasteurized milk^13^. Research across various food categories revealed a link between hypervirulent *L. monocytogenes* clones (CC1, CC4, and CC6) and dairy products produced from raw milk^10^. However, no studies evaluated the growth behavior of outbreak-related *L. monocytogenes* and its isogenic derivatives overexpressing key virulence factors in UHT and raw milk. The most important virulence factors of *L. monocytogenes* strains are encoded in the *inlA-inlB* loci and the pathogenicity islands LIPI-1, LIPI-3, and LIPI-4^8^. LIPI-1 and the *inlA-inlB* locus are regulated by the transcriptional activator PrfA and form part of the core genome, whilst LIPI-3 is part of the accessory genome^4^. Genetic analysis of 6,633 food and clinical isolates of *L. monocytogenes*, in conjunction with epidemiological and clinical data, identified full-length InlA and LIPI-3 as being strongly associated with infectious potential^9^. Moreover, listeriosis clinical cases have been associated with lineage I strains^9^. The role of virulence determinants in food contamination has been somewhat overlooked, as most studies primarily focus on their role in microbial pathogenesis. Consequently, whether PrfA-controlled genes and LIPI-3 affect the performance of hypervirulent lineage I *L. monocytogenes* in UHT and raw milk remained experimentally unconfirmed. Here, we analysed the fitness consequences of overexpressing virulence traits (LIPI-3 and the PrfA regulon) using a CC1 lineage I *L. monocytogenes* strain related to a cheese outbreak^11^. Using LIPI-3 and PrfA-controlled genes to be constitutively activated, we demonstrate that LIPI-3 overexpression, compared to PrfA *o*verexpression, enhances the listerial growth rate in UHT and raw milk at refrigeration temperature. This fitness advantage conferred by LIPI-3 could impose a beneficial cost outside the host and provide new insights into understanding why LIPI-3 is frequently found in CC1, CC4, and CC6 isolates, which are also overrepresented in dairy products^10^. On the contrary, PrfA overexpression imposed a significant cost to *L. monocytogenes* in UHT and raw milk, which is in agreement with Vasanthakrishnan et al. (2015), who previously demonstrated experimentally that PrfA overexpression lowers *L. monocytogenes* fitness in BHI medium and soil^35^. Similar to our results related to PrfA constitutive activation, a study on *Salmonella* associated with the cost of virulence factors in in vitro culture showed that expression of the type III secretion system (TTSS)-1 was related to growth retardation^36^. Contamination with non-pathogenic *Listeria* spp., *L. ivanovii*, or virulence-overexpressing *L. monocytogenes* did not alter the raw milk microbiome, maintaining a stable community throughout the study, which was dominated by psychrotrophic genera such as *Pseudomonas* and *Carnobacterium*. Indeed, *Pseudomonas* has been consistently reported as a dominant taxon in refrigerated milk^37^ and identified as a member of the core raw milk microbiota in a recent large-scale survey^38^. The occurrence of *Carnobacterium* in raw milk across different breeds, together with functional traits such as bacteriocin production, has been previously reported^39,40^. Therefore, the observed stability of the raw milk microbiota suggests that these psychrotrophic genera play a central role in shaping the community dynamics of this type of food matrix.

In conclusion, our study shows that at 4 °C: (i) the PrfA* mutant exhibits a fitness cost in milk, reflected in its reduced growth rate compared to the epidemic *L. monocytogenes* F2365 wild type strain; (ii) constitutive expression of LLS does not significantly impact growth parameters compared to the epidemic *L. monocytogenes* F2365 wild type strain; (iii) *L. ivanovii*, *L. innocua*, and *L. valentina* are not suitable surrogates for the epidemic *L. monocytogenes* F2365 strain in UHT and raw milk; and (iv) neither the overexpression of virulence traits in epidemic *L. monocytogenes* F2365 strain nor the characteristics of non-pathogenic *Listeria* species altered the dynamics of microbial communities in raw milk.

Our results regarding the use of *Listeria* spp. as index or indicator organisms will help food-processing facilities identify conditions that increase the risk of *L. monocytogenes* presence, growth, and/or contamination. This knowledge will help to reduce the number of human listeriosis cases caused by food contamination with *L. monocytogenes*.

## Methods

### Bacterial strains and inoculum preparation

Six *Listeria* spp. isolates were used in this study, comprising both pathogenic (*L. monocytogenes* (n = 3) and *L. ivanovii*) and non-pathogenic strains (*L. innocua* and *L. valentina*). Detailed strain information is provided in Table S4. The *L. monocytogenes* F2365 wild type (WT) strain was selected for its epidemiological significance, as it was responsible for the 1985 listeriosis outbreak linked to Mexican-style cheese, which resulted in 142 human cases and 48 deaths^11^. To achieve constitutive activation of PrfA, we used the *L. monocytogenes* F2365_PrfA* strain, which carries a Gly145Ser substitution in this transcriptional activator, leading to the continuous expression of LIPI-1 virulence factors^14,41^. Additionally, to assess the role of LLS in growth within UHT and raw milk, we employed *L. monocytogenes* F2365_LLS (previously denominated pHELP:llsA, a synthetic strain designed to constitutively express LLS by placing the LIPI-3 genes under the control of the strong constitutive pHELP promoter^16^.

All isolates were stored at −80 °C in glycerol stocks. Before use, strains were streaked onto Brain Heart Infusion (BHI) agar and incubated at 37 °C for 48 h. A single colony was aseptically transferred to 2 mL of BHI broth and grown at 37 °C for 16 h with shaking at 250 rpm. Cultures were then resuspended in phosphate-buffered saline (PBS) to achieve a final inoculum of approximately 2.3 Log₁₀ CFU/mL ( ~ 200 CFU/mL).

### Milk sample collection and microbiological assessment

To assess *Listeria* spp. growth in UHT milk, four 1 L containers of the same commercial brand were purchased locally. Raw bovine milk was collected under refrigeration 24 h post-milking from the Milk Analysis Laboratory (Polytechnic University of Valencia) and processed immediately upon arrival. *Listeria* spp. detection and MAB counts were performed as previously described^25,42^ following the ISO 11290-1^43^, and ISO 11290-2^44^ for *Listeria* spp., and according to ISO 4833-2^45^ for quantification of MAB. The initial MAB count at 4 °C was 9,400 CFU/mL (3.97 Log₁₀CFU/mL).

### Assessment and modelling of *Listeria* growth in milk matrices

Bacterial growth was monitored at 4 °C for up to 43 days in UHT milk and 35 days in raw milk. *Listeria* spp. was enumerated on BHI agar (for UHT milk) or ALOA agar (for raw milk), while MAB were quantified on Plate Count Agar (PCA). Samples with ≤100 CFU/mL were directly plated; otherwise, serial dilutions in PBS were performed. Plates were incubated at 37 °C for 24 h (*Listeria* spp.) and 30 °C for 72 h (MAB). Each experiment included four biological replicates per isolate and matrix, with three technical replicates per time point. Log₁₀CFU/mL values and pH (measured with a sensION™+ pH meter and XS SEMI-MICRO electrode) were recorded at each sampling day.

The growth of *Listeria* spp. over time was modeled using the modified Gompertz equation^46^, a widely used primary model that describes bacterial population dynamics as a function of time. As an empirical sigmoidal function, it enables reliable estimation of kinetic parameters such as maximum growth rate (μ_max_), lag time (λ), and asymptotic population size (C). Although the Baranyi model^47^ offers a mechanistic approach better suited to fluctuating environmental conditions, the Gompertz equation has been widely validated for constant environments^48,49^ and frequently applied to model *L. monocytogenes* growth in refrigerated dairy products^50–52^. Given that the present experiments were conducted under static conditions, the Gompertz model was considered the most appropriate choice.

The equation used was:

$${\mathrm{Log}}_{10}{\rm{N}}({\rm{t}})={\mathrm{Log}}_{10}{\mathrm{N}}_{0}+\begin{array}{c}{\mathrm{C}}* \end{array}\exp \left(-\exp \left(\left(\frac{\begin{array}{c}2.718* \end{array}{{\rm{\mu }}}_{\max }}{\mathrm{C}}\right)* \left(\lambda -\mathrm{t}\right)+1\right)\right)$$

Where *N*(t) represents *Listeria* spp. concentration (CFU/mL) at a particular time; *N*_*0*_ the initial *Listeria* spp. concentrations (CFU/mL); *C* the difference between the curve asymptotes (Log_10_(CFU/mL)), corresponding to the difference between the maximum and the minimum Log_10_ CFU/mL reached; μ_max_ the maximal growth rate (Log_10_(CFU/mL)/day); λ the lag phase (days); and t the particular time (days).

Initial estimates for N₀ of each biological replicate were derived by plotting experimental Log_10_ CFU/mL data against time. Kinetic parameters were fitted using Statgraphics Centurion XVII software. The goodness of fit was evaluated by calculating the Root Mean Square Error (RMSE), which reflects the deviation of model predictions from observed values, and the adjusted coefficient of determination (R²adj), which indicates the proportion of variance explained by the model. The goodness-of-fit indicators obtained in our study demonstrated that the model reliably described the data: all the RMSE values were <0.259 and <0.157 for UHT and raw milk, respectively, and the adjusted R²_adj_ values > 0.981 and >0.860 for UHT and raw milk, respectively, confirming its accuracy in capturing the growth kinetics of *Listeria* spp. in UHT and raw milk (Table 1).

### DNA extraction, library preparation, and 16S rRNA sequencing of raw milk samples

Metagenomic analysis was conducted on raw milk samples collected at days 14 and 28, corresponding to the exponential and stationary phases of *Listeria* spp., to assess microbiota dynamics. DNA was extracted using the DNeasy PowerLyzer PowerSoil Kit (Qiagen) using Tissue lyser II (Qiagen) at 30 Hz, 10 min, 4 °C. Amplification targeted the V3–V4 region of the 16S rRNA gene using specific primers. PCR products were purified with AMPure XP beads and dual-indexed using Nextera XT v2 adapters in a second PCR. Libraries were normalized (SequalPrep kit (ThermoFisher Scientific)), quantified by qPCR, pooled, and sequenced on an Illumina MiSeq platform (2 × 300 bp, v3 chemistry, 10 pM loading, 15% PhiX spike-in). To ensure data integrity, negative controls were analyzed. Amplicon data were processed in QIIME2 (v2019.4) using DADA2 for quality filtering, denoising, and amplicon sequence variant calling (ASV)^53,54^. Reads were truncated at Q20 (299 nt forward, 243 nt reverse); average sample size was 83,230 reads with 8,428 ASVs detected. ASVs were aligned using the qiime alignment mafft method^55^. The alignment was used to create a tree and to calculate phylogenetic relations between ASVs using qiime2 phylogeny fasttree method^56^. Diversity metrics (richness, Pielou’s evenness) and Weighted UniFrac distances were calculated after rarefaction to 49,400 reads. The smallest sample size was chosen for subsampling^57^. Taxonomy was assigned using a Bayesian Classifier trained with Silva database (i.e., 99% ASVs database) using the qiime feature-classifier classify-sklearn method^58^. Phylotypes were filtered to discard contaminant Eukariota DNA-derived amplicons using Blast against the mentioned database with a 90% identity cutoff. Mock community and negative controls were processed the same way as samples.

### Statistical analysis

Statistically significant differences in *Listeria* spp. and MAB growth, and taxon abundance were assessed using ANOVA or Kruskal–Wallis tests. Alpha diversity was analyzed using generalized mixed models: negative binomial for Richness and beta regression for Evenness. Beta diversity (Weighted UniFrac analysis) was compared using PERMANOVA, ANOSIM, and PERMDISP. Taxon differential abundance was evaluated with Kruskal–Wallis’s test. For all statistical analyses, the significance level was set at 0.05. Figures were generated with GraphPad Prism (Version 8.0) and Adobe Illustrator (Version 27.0).

## Supplementary information

Supplementary information

## Data Availability

The datasets generated and analysed during the current study are available in the BioProject repository, under BioProject number: PRJEB94572.
