# Supplementary material for: Impact of virulence factors overexpression on Listeria monocytogenes F2365 epidemic strain fitness and the limitations of surrogate species in UHT and raw milk
Source: NPJ Sci Food. 2025 Dec 15;10:15. doi: 10.1038/s41538-025-00658-7 (PMC12816694; doi:10.1038/s41538-025-00658-7)
Supplement: Supplementary file 1 — Supplementary information [file 41538_2025_658_MOESM1_ESM.pdf]

Table S1. pH values of the four biological replicates of each isolate of *Listeria* spp. in UHT milk at 4 ºC. T0, T3, T5, T7, T10, T14, T17, T21, T25, T29, T35, and T43 indicate the monitored days of the study.

**pH values of biological replicate samples**	**Monitored days of the study**											
	**T0**	**T3**	**T5**	**T7**	**T10**	**T14**	**T17**	**T21**	**T25**	**T29**	**T35**	**T43**
F2365 WT	-	6.74	6.83	6.82	6.9	6.9	6.85	6.88	6.85	6.79	6.76	6.75
F2365 WT	-	6.75	6.8	6.74	6.92	6.89	6.84	6.87	6.83	6.76	6.73	6.72
F2365 WT	-	6.75	6.76	6.76	6.91	6.87	6.84	6.85	6.81	6.75	6.73	6.72
F2365 WT	-	6.74	6.79	6.74	6.89	6.82	6.83	6.84	6.81	6.75	6.72	6.72
F2365_PrfA*	-	6.73	6.82	6.74	6.9	6.85	6.86	6.86	6.84	6.79	6.76	6.74
F2365_PrfA*	-	6.75	6.81	6.74	6.89	6.85	6.83	6.88	6.84	6.79	6.75	6.75
F2365_PrfA*	-	6.73	6.77	6.75	6.9	6.85	6.84	6.86	6.84	6.8	6.75	6.76
F2365_PrfA*	-	6.76	6.79	6.75	6.9	6.84	6.84	6.87	6.86	6.81	6.77	6.76
F2365_LLS	-	6.76	6.82	6.75	6.89	6.84	6.83	6.83	6.8	6.74	6.7	6.71
F2365_LLS	-	6.75	6.8	6.74	6.9	6.82	6.82	6.83	6.79	6.74	6.7	6.7
F2365_LLS	-	6.74	6.79	6.74	6.87	6.82	6.82	6.83	6.81	6.74	6.69	6.7
F2365_LLS	-	6.73	6.79	6.74	6.88	6.81	6.83	6.84	6.81	6.75	6.7	6.71
L. innocua	-	6.75	6.8	6.75	6.89	6.83	6.82	6.83	6.79	6.71	6.68	6.71
L. innocua	-	6.79	6.79	6.75	6.89	6.83	6.83	6.83	6.81	6.74	6.75	6.71
L. innocua	-	6.77	6.77	6.75	6.9	6.81	6.81	6.85	6.82	6.76	6.79	6.7
L. innocua	-	6.75	6.77	6.74	6.9	6.81	6.82	6.84	6.84	6.77	6.8	6.69
L. valentina	-	6.75	6.8	6.81	6.82	6.8	6.83	6.85	6.84	6.81	6.86	6.77
L. valentina	-	6.78	6.74	6.78	6.77	6.8	6.82	6.86	6.84	6.83	6.92	6.82
L. valentina	-	6.74	6.7	6.73	6.79	6.79	6.81	6.9	6.84	6.81	6.87	6.83
L. valentina	-	6.76	6.82	6.73	6.76	6.78	6.81	6.89	6.85	6.81	6.87	6.83
L. ivanovii	-	6.76	6.79	6.79	6.84	6.8	6.78	6.84	6.83	6.76	6.8	6.76
L. ivanovii	-	6.76	6.82	6.77	6.85	6.8	6.81	6.86	6.84	6.79	6.8	6.75
L. ivanovii	-	6.77	6.79	6.74	6.82	6.81	6.82	6.84	6.84	6.8	6.82	6.76
L. ivanovii	-	6.76	6.8	6.77	6.8	6.8	6.81	6.85	6.84	6.8	6.81	6.76
**Not inoculated sample**	6.82											

Table S2. pH values of the four biological replicates of each isolate of *Listeria* spp. in raw milk at 4 ºC. T0, T3, T5, T7, T11, and T35 indicate the monitored days of the study.

**pH values of biological replicate samples**	**Monitored days of the study**					
	**T0**	**T3**	**T5**	**T7**	**T11**	**T35**
F2365 WT	6.88	6.89	6.97	6.91	6.88	6.23
F2365 WT	6.88	6.92	7	6.93	6.93	6
F2365 WT	6.93	6.91	6.99	6.95	7	6.19
F2365 WT	6.86	6.9	7	6.91	6.93	6.14
F2365_PrfA*	6.86	6.9	6.99	6.86	6.89	6.23
F2365_PrfA*	6.86	6.91	7.01	6.88	6.92	6.22
F2365_PrfA*	6.86	6.9	6.97	6.9	6.91	6.27
F2365_PrfA*	6.84	6.91	7.03	6.9	6.79	6.18
F2365_LLS	6.85	6.88	6.96	6.89	6.94	6.05
F2365_LLS	6.85	6.88	6.97	6.89	6.93	6.16
F2365_LLS	6.87	6.88	6.95	6.87	6.78	6.16
F2365_LLS	6.84	6.9	6.95	6.9	6.9	6.11
L. innocua	6.84	6.89	6.94	6.89	6.95	6.27
L. innocua	6.86	6.88	6.96	6.94	6.94	6.19
L. innocua	6.85	6.91	6.96	6.91	6.88	6.24
L. innocua	6.83	6.89	6.98	6.87	6.93	6.19
L. valentina	6.85	6.9	6.97	6.88	6.84	6.22
L. valentina	6.87	6.88	6.96	6.86	6.85	6.23
L. valentina	6.84	6.86	6.98	6.86	6.85	6.2
L. valentina	6.87	6.87	7.01	6.87	6.85	6.3
L. ivanovii	6.85	6.87	6.97	6.87	6.83	6.26
L. ivanovii	6.83	6.88	6.97	6.87	6.84	6.19
L. ivanovii	6.85	6.89	6.97	6.89	6.88	6.26
L. ivanovii	6.85	6.88	6.95	6.9	6.83	6.16
**Not inoculated sample**	6.86					

Table S3. Estimated kinetic parameters of mesophilic aerobic bacteria grown in raw milk at 4 ºC obtained from the modified Gompertz model.

Kinetic parameter	Mesophilic aerobic bacteria (MAB)					
	△F2365	△F2365_PrfA*	△F2365_LLS	△*L. innocua*	△*L. valentina*	△*L. ivanovii*
λ (days)	0.04 ± 0.09 a	0.00 ± 0.00 a	0.01 ± 0.02 a	0.09 ± 0.20 a	0.13 ± 0.26 a	0.11 ± 0.23 a
μ_{max} [Log_{10}(CFU/mL)/day]	0.29 ± 0.01 a	0.30 ± 0.01 a	0.30 ± 0.02. a	0.31 ± 0.05 a	0.30 ± 0.02 a	0.32 ± 0.05 a
$Log_{10}Nf_{max}$ [Log_{10}(CFU/mL)]	9.40 ± 0.11. a	9.47 ± 0.03 a	9.54 ± 0.14 a	9.31 ± 0.31 a	9.65 ± 0.21 a	9.12 ± 0.50 a
RMSE	0.336	0.311	0.355	0.360	0.303	0.277
R^2adj.	0.965	0.971	0.964	0.962	0.972	0.975

Data represents estimated parameter ± standard deviation. Values in the same row followed by the same lowercase letter are not significantly different ($P > 0.05$). Parameters are estimated from the growth curves and each point of the curves was based on four replicate samples in UHT milk and raw milk.

Table S4. *Listeria spp.* strains used in this study.

Strain	Lineage	CC	References	ID number
L. monocytogenes F2365 (WT)	I	CC1	Linnan MJ et al. 1988	BUG 3012
L. monocytogenes F2365_PrfA*	I	CC1	Quereda JJ et al. 2018	BUG 3651
L. monocytogenes F2365_LLS	I	CC1	Quereda JJ et al. 2016	BUG 3817
L. innocua			Glasser et al. 2001	BUG 499
L. valentina			Quereda JJ et al. 2020	Lisval 106
L. ivanovii subs. ivanovii			Palacios-Gorba et al. 2021	Lisval 449
